# Supplementary material for: Significant Broad-Spectrum Antiviral Activity of Bi121 against Different Variants of SARS-CoV-2
Source: Viruses. 2023 May 31;15(6):1299. doi: 10.3390/v15061299 (PMC10305169; doi:10.3390/v15061299)
Supplement: Supplementary file 1 [file viruses-15-01299-s001.zip › viruses-2384651-supplementary.pdf]

Supplemental figure

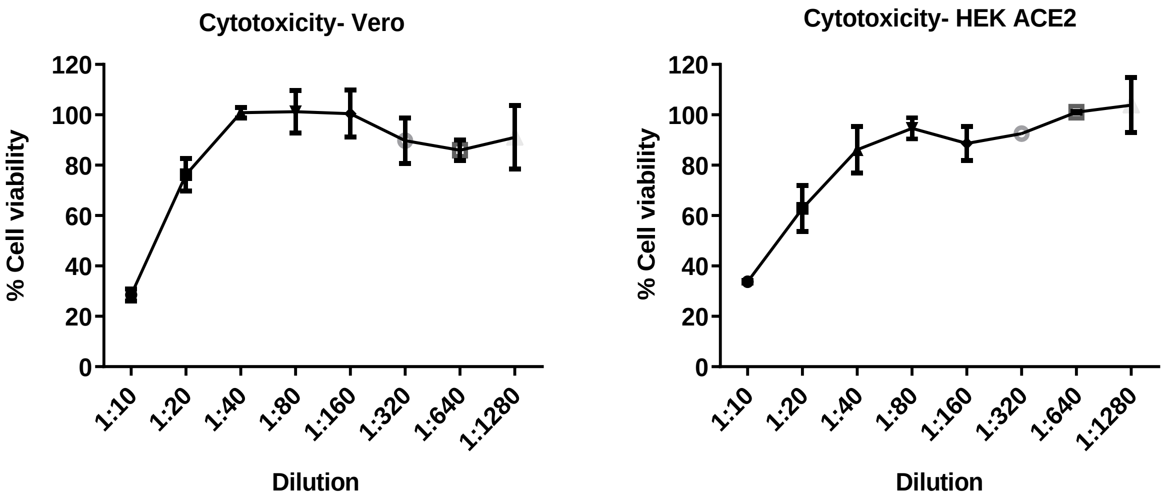

Figure S1a. Cytotoxicity of Bi121 was measured in VeroE6 and HEK-ACE2 cells by MTT assay.

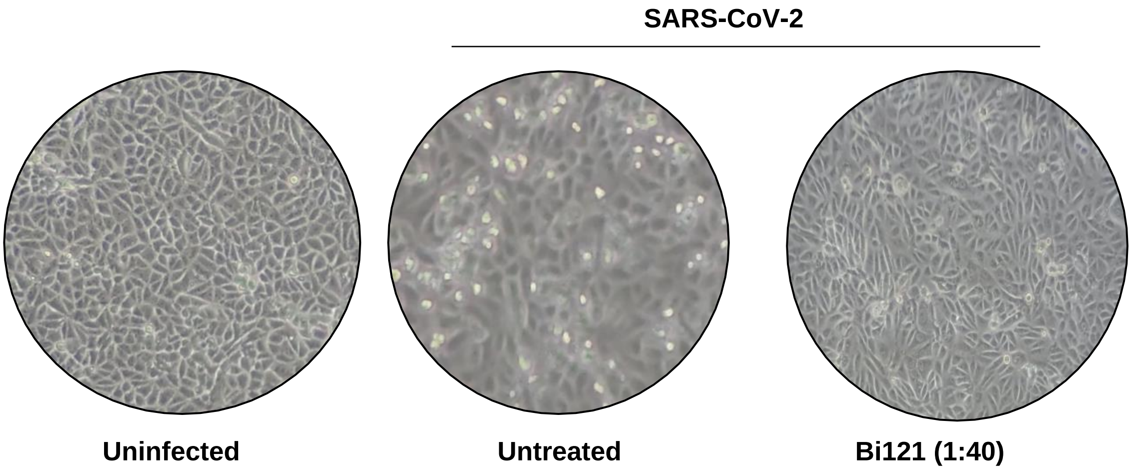

Figure S1b. Representative well images of SARS-CoV-2 infected VeroE6 cells in untreated and Bi121 treated
